# Geospatial analysis of short sleep duration and cognitive disability in US adults: a multi-state study using machine learning techniques

**DOI:** 10.1186/s13040-025-00456-7

**Published:** 2025-06-13

**Authors:** Tue T. Te, Alex A. T. Bui, Constance H. Fung, Mary Regina Boland

**Affiliations:** 1 Department of Medicine, David Geffen School of Medicine at University of California, Los Angeles (UCLA), Los Angeles, CA USA; 2 Geriatric, Research, Education and Clinical Center, VA Greater Los Angeles, Los Angeles, CA USA; 3 Medical and Imaging Informatics Group, Department of Radiological Sciences, University of California, Los Angeles, Los Angeles, CA USA; 4 Department of Data Science, Herbert W. Boyer School of Natural Sciences, Mathematics, and Computing, Economics and Government, Alex G McKenna School of Business, Saint Vincent College, Mathematics, Latrobe, PA USA

## Abstract

**Background:**

There is evidence of increased risk of cognitive disability due to short sleep duration and adverse Social Determinants of Health (SDoH). To determine whether spatial associations (correlation between spatially distributed variables within a given geographic area) exist between neighborhoods with short sleep duration and cognitive disability across the United States (US) after adjusting for other factors. We conducted a spatial analysis using a spatial lag model at the neighborhood-level with the census tract as unit-of-analysis within each state in the US. We aggregated our results nationally using a weighted analysis to adjust for the number of census tracts per state. This study used Centers for Disease Control and Prevention (CDC) data on short sleep duration, cognitive disability and other health factors. We used 2021–2022 neighborhood-level data from the CDC and US Census Bureau adjusting for social determinants of health (SDoH) and demographics, excluding Florida due to inconsistencies in data availability. Our exposure variable was self-reported short sleep defined by the CDC (“sleep less than 7 hours per 24 hour period”). Our outcome was self-reported cognitive disability defined by the CDC (“difficulty concentrating, remembering, or making decision”). We adjusted for other factors including ‘health outcomes’, ‘preventive practices’, and the CDC’s Social Vulnerability Index.

**Results:**

The spatial analysis revealed a significant association between short sleep duration and an increased risk of cognitive disability across the US (estimate range [0.29; 1.27], *p* < 0.005) after adjustment. Notably, six Western states (New Mexico, Alaska, Arizona, Nevada, Idaho, and Oregon) were at increased risk of cognitive disability due to short sleep duration and this pattern was significant (*p* = 0.007).

**Conclusions:**

Our study highlights the importance of short sleep duration as a significant predictor of cognitive disability across the US after adjusting for other confounders. The association between short sleep and cognitive disability was especially strong in the Western region of the US providing a deeper understanding of how geographic context and local factors can shape health outcomes.

**Supplementary Information:**

The online version contains supplementary material available at 10.1186/s13040-025-00456-7.

## Background

Cognitive disability (CD), often characterized by difficulty with memory, attention, decision-making, and other cognitive functions, is a growing concern, particularly among older adults [1]. Numerous studies have linked CD to a variety of risk factors, including aging, genetic predisposition, and chronic health conditions such as hypertension, diabetes, and cardiovascular disease [1, 2]. In recent years, attention has increasingly turned to lifestyle factors, particularly sleep duration, as contributors to cognitive health [3]. The pattern of CD in the United States (US) shows significant variation, particularly among minority racial and ethnic groups such as Black and Hispanic individuals [4]. This disparity is influenced by several factors, including socioeconomic status, access to healthcare, and chronic stress [5, 6]. Garcia et al. found that older Black and Hispanic adults are more likely to be cognitively impaired than older White counterparts. Educational disadvantages contribute significantly to those disparities [7].

Increased CD risk has been linked to short sleep duration– less than 7 h within a 24-hour day [8]– and other conditions such as obesity, diabetes, hypertension, heart disease, stroke, and depression [9, 10, 11]. Socioeconomic factors were key determinants of inadequate sleep as reported by about one-third of US adults in a 2014 survey [12]. Pronounced sleep duration disparities have persisted over decades, particularly among Blacks and Hispanics compared to Whites [13, 14, 15]. Geography significance is highlighted in recent studies showing inadequate sleep most prevalent in the US Southeast and Appalachian regions [12, 16].

While research has explored the link between short sleep and CD, as well as the role of social determinants of health (SDoH) in each, little is known about how SDoH and geographic factors interact to influence this relationship. There are several gaps in the previous literature that limit our understanding of how short sleep duration and CD are connected in depth in the context of SDoH. One key issue is the heterogeneity of study designs, which makes it difficult to compare results across studies or conduct meta-analyses. Differences in sample sizes, methods of assessing sleep and cognitive function, and failure to adequately control for confounders such as comorbidities, lifestyle factors, and socioeconomic status further complicate the big picture [17, 18]. Additionally, studies often focus on specific populations or regions, limiting the generalizability of their findings. For example, some studies have found associations between short sleep and CD in specific ethnic groups or specific regions, but these results may not apply universally [19, 20].

This study aims to fill these gaps by investigating the relationship between short sleep duration and CD across the entire US, adjusting for other health conditions and key SDoH factors, which can confound this relationship. Using a nationwide dataset from the Centers for Disease Control and Prevention (CDC) [21] and the US Census Bureau, [22] we conducted spatial analysis including Moran’s I and autocorrelation regression modeling to assess the effects of short sleep on CD at the neighborhood-level, adjusted for several potential confounders. Additionally, weighting our analysis by the number of census tracts per state to compare regions across the US was used to examine regional variations in the impact of short sleep on CD. Understanding these dynamics will help guide public health interventions aimed at reducing sleep disparities and mitigating the risk of CD, particularly in vulnerable populations. Ultimately, these findings could contribute to healthier aging across diverse communities by addressing the broader social and environmental factors that influence CD.

## Methods

### Datasets

We used the CDC Population Level Analysis and Community Estimates (CDC PLACES) [21] and the CDC Social Vulnerability Index (CDC SVI) [22] at the census-tract levels. CD was assessed via self-report using the item: “Because of a physical, mental, or emotional condition, do you have serious difficulty concentrating, remembering, or making decisions?” *(Yes/No).* Sleep duration was measured with the question: “How many hours of sleep do you get on average in a 24-hour period?”, with responses of < 7 h classified as ‘*short sleep duration*’. The number of hours of sleep was recorded as a whole number (rounding up for *≥* 30 min and rounding down for < 30 min). The rounding rule that was applied is consistent with the methodology used by the CDC in the Behavioral Risk Factor Surveillance System (BRFSS), which is a primary data source for the CDC PLACES data [23].

The selection of health, socioeconomic, and demographic covariates was guided by existing literature on the bidirectional associations between short sleep duration and various chronic conditions such as hypertension, diabetes, depression, and obesity [9]. In addition, socioeconomic and demographic factors—such as income, education, race/ethnicity, and housing quality—are known to influence both cognitive health and sleep issues [24, 25]. We retrieved health condition estimates from the CDC PLACES, [21] selecting only those measures with the least amount of missing data across the US for comprehensive coverage.

Among the 84,414 census-tracts in the US, 68,172 (80.76%) census tracts had CDC PLACES information [26]. We included all census tracts with CDC PLACES information. The 2010 geographic shapefiles of census tracts for 50 US states including District of Columbia (DC) were downloaded from the US Census Bureau’s Cartographic Boundary Files and used to map and visualize the census tract-level data [27]. We used CDC PLACES accessed via Github, [28] and *sf* package (version 1.0–18) [29] in R (version 4.3.3.).

Additional detail is in our Additional Method.

### Spatial analysis

#### Moran’s i and spatial autocorrelation

We first used Moran’s I test to determine if there was any spatial correlation in each state using census-tract level data. We did this for CD and sleep only (without controlling for any health/socioeconomic factors). Moran’s I test requires a spatial interaction matrix, and to create this matrix, we used the adjacency of tracts (which we will calculate also for the next step) as identified via neighboring polygons based on shared boundaries. The spatial interaction matrix was calculated from an existing matrix (*w)*. Then, the transpose (*t(w))* was added and we subtracted the element-wise product of *w* and *t(w).*

#### Spatial weight matrix construction

S*pdep* (version 1.3-6) [30] and *spatialreg* (version 1.3-5) [31] packages in R were used to create a spatial adjacency matrix using neighborhood information derived from shapefiles. We used contiguity-based spatial weights rather than distance-based weights because our spatial units of analysis were census tracts, which vary in size and shape across states. Contiguity-based weights allow for a consistent definition of neighborhood structure, especially in heterogeneous geographic areas. This approach is commonly used in public health spatial analyses to capture both direct and corner-based tract interactions [26]. This constructs a spatial weight matrix based on contiguity using *poly2nb()* with *queen* contiguity, which defines neighboring census tracts as those that share either a border or a vertex. The adjacency of tracts was determined by identifying neighboring polygons based on shared boundaries. We converted the neighborhood list to a spatial weights object using *nb2listw().* We set *true* for *zero policy* option to account for census tracts that have no neighbors, ensuring that all tracts were included in the model.

#### Spatial analysis model specification

The *spatial lag* models were constructed separately for each state; therefore, we conducted a model for each state. These models incorporated lagged values of neighboring tracts’ CD as the outcome and short sleep duration as a main predictor, adjusting for covariates from the CDC PLACES in prevalence of health outcomes, prevention practices, health risk behaviors, disabilities, health statuses, and community factors; and from the CDC SVI for socioeconomic and income, education, employment, housing quality, transportation access, and racial/ethnic composition factors within each state. The coefficients, p-values, and confidence intervals (CI) were extracted from these models.

#### State-level weighted forest plot

We weighted our analysis by the number of census-tracts per state to generate a state-level weighted result. The mean estimates for each state were incorporated into forest plot visualizations. The weighted estimates were also summarized at the national level by computing a mean estimate across all states.

#### Choropleth-like visualization

We visualized the relationship between CD and short sleep duration along with various health and socioeconomic variables across US states using choropleth and heatmap techniques. We plotted estimates and CIs for the state with the highest estimate (highest risk of CD) and the state with the lowest estimate displaying all health conditions and socio-economic factors using *ggplot2* package (version 3.5.1) [32] in R.

### Explore relationship per region in state by state-level weighted analysis meta-analysis

We grouped states into four US Census Bureau regions: Northeast (9 states), Midwest (12 states), South (17 states), and West (13 states) [33]. We selected states with model estimates showing > 100% increase in risk of CD (those with strong associations) after adjustment from our adjusted spatial analysis models to determine if those states were disproportionality in certain regions in the US. We used the Fisher’s exact test to determine statistical significance.

## Results

### Overview of dataset and variables

#### Dataset overview

We included all US census tracts (84,414) and then removed those without data from CDC PLACES (maximum number of census tracts with CDC PLACES is 72,337), Census Bureau and CDC SVI resulting in a final set of 57,404 census tracts from across the US. These represent the most densely populated areas in the US. In Table 1, California has the most census tracts (6,809), followed by New York (4,311); while Wyoming (103) and Alaska (143) have the fewest. The South and Midwest have higher percentages of CD, with Mississippi reporting the highest median (interquartile range [IQR]) at 19.4%(IQR = 16.6%, 22.9%), followed by Louisiana at 18.9% (IQR = 15.4%,22.2%); in contrast, Hawaii has the lowest median at 9.7% (IQR = 8.1%,11.1%), followed by Nebraska at 10.3% (IQR = 9.0%,12.1%). Hawaii has the highest median of short sleep duration at 39.85% (IQR = 37.6%, 42.2%), followed closely by West Virginia at 39.75% (IQR = 37.9%, 42.2%); while South Dakota has the lowest medians at 27.9% (IQR = 26.6%, 29.4%), and Minnesota at 28.0% (IQR = 25.8%, 29.8%).

**Table 1:** Demographics of the dataset

State	Total census tracts	Cognitive Disability (%)		Short Sleep Duration (< 7 h) (%)		State	Total census tracts	Cognitive Disability (%)		Short Sleep Duration (< 7 h) (%)		
		Median(IQR)	Range	Median(IQR)	Range			Median(IQR)	Range	Median(IQR)	Range	
Northeast						West						
Connecticut	766	10.5(8.7, 13.7)	5.3, 32.2	33(30.8,36.3)	25.8,49.9	Alaska	143	12.9(11.2,14.8)	7.2,26.7	32.3(30.6,33.5)		25.8,38.1
Massachusetts	1307	12.3(10.2, 15.8)	6.5, 36.3	31(28.3,34)	23.5,45.1	Arizona	1280	13.9(10.8,18.3)	6.8,34.9	32.7(30.2,35.3)		22.3,47.1
Maine	305	14.9(12.7, 16.8)	7.8, 30.5	32.1(30.1,33.7)	25.4,40	California	6809	12.5(9.6,16.4)	5.1,35.2	32.4(29.7,34.9)		19.8,47.6
New Hampshire	234	11.5(10.1, 13.2)	6.8, 25.1	31.8(30.1,33.4)	25.7,39.9	Colorado	1046	11.4(9.3,14.1)	5.9,36.6	28.3(26,30.4)		21.6,43.9
New Jersey	1820	10.8(8.8, 13.7)	5.4, 29.9	34(31.4,36.7)	24.3,50.5	Hawaii	194	9.8(8.1,11.1)	5.2,23.1	39.9(37.6,42.2)		32.9,52.9
New York	4311	12(9.9, 14.8)	5.5, 36.6	33.9(31.6,37.4)	23.2,49.9	Idaho	187	15.3(13.7,17.8)	8.2,32.9	30.7(29.6,32.3)		24.2,38.7
Pennsylvania	2915	13.3(11.1, 15.9)	6.2, 37.0	35.2(33.1,37.4)	22.8,52.7	Montana	216	13.1(11.6,14.7)	6.9,26	30.2(28.9,31.8)		20.7,40.0
Rhode Island	234	12(10.4, 15)	6.5, 25.5	34.7(31.9,37.1)	25.6,43	New Mexico	391	15.8(12.7,19)	7.4,40.2	32(30.2,34.1)		20.3,43.1
Vermont	164	12.1(10.5, 13.5)	7.4, 22.0	28.7(26.1,32.3)	22.6,36.6	Nevada	593	14.7(12.2,18.5)	7.5,30.1	35.5(33.5,37.7)		25.4,45.9
South						Oregon	666	14.3(12.3,16.8)	7.6,31.3	28.8(26.9,30.5)		21.4,37.4
Alabama	930	16.8(14.2,19.5)	6.9,36.3	39.2(36.3,43.4)	27,54.2	Utah	476	13.4(11.3,15.7)	7.4,33	30.7(28.8,32.9)		24.1,41.9
Arkansas	545	17.9(15.6,20.4)	7.5,30.8	34.3(32.5,37)	25.7,47.7	Washington	1146	12.6(10.6,14.8)	6.4,31.5	31.1(28.4,33.1)		22.2,42.6
DC*	154	10.9(8.2,15.6)	5.6,27.8	33.8(28.8,40.5)	23.6,45.6	Wyoming	103	13(11.8,14.3)	8.9,26	30.8(29.8,33)		25.5,37.4
Delaware	175	13(10.3,14.9)	6.3,27.7	33.8(30.7,36.8)	23.6,47.6	Midwest						
Georgia	1243	16.1(12.8,18.9)	6.5,36.3	37.2(34,41.4)	25.7,53.8	Iowa	1313	11.3(10.3,13)	6.3,26.5	30.5(29.3,32)		23.2,44.9
Kentucky	908	18.7(15.2,22.1)	7.7,40.7	38(35.4,39.8)	26.8,51.1	Illinois	2964	14(11.3,17.3)	6.4,40.0	32.3(30.3,34.9)		23.2,49.5
Louisiana	848	18.9(15.4,22.2)	7.7,34.7	37.8(34.7,42.4)	24.6,50.7	Indiana	763	14.2(12.1,17.1)	6.6,34.8	34.8(32.9,37.2)		24.4,51.0
Maryland	1265	11.0(9.0,13.3)	5.4,35.2	33.5(30.2,39.1)	20.3,49.8	Kansas	646	13.3(11.4,16.2)	5.8,29.9	32.5(30.3,34.9)		23.0,49.6
Mississippi	1131	19.4(16.6,22.9)	8.2,33.8	35.1(32.2,38.9)	23.4,48.1	Michigan	2443	12.7(10.5,15.8)	5.7,35.6	33.8(31.9,37)		25.3,52.7
North Carolina	1694	14.3(11.7,17.2)	6.2,34.7	32.9(30.3,36.1)	23.1,50	Minnesota	1167	11.3(9.6,12.8)	6.3,27.7	28(25.8,29.8)		20.1,40.5
Oklahoma	866	16.5(13.9,19.2)	7.2,32.4	33.9(31.8,35.7)	23.4,47.9	Missouri	452	16.1(13.2,19.1)	7.2,41.6	33.8(31.8,36.2)		24.7,50.6
South Carolina	875	14.8(11.9,17.6)	5.6,35.6	35.5(32.5,38.8)	25,48.6	North Dakota	164	11.2(10.4,12.8)	7.1,25.5	29.4(28.1,30.8)		24.8,39.9
Tennessee	1270	16.8(14.1,19.4)	7.3,36.7	35.2(33,37.3)	24.1,49.6	Nebraska	501	10.3(9,12.1)	5.5,24.0	28.5(27,30.6)		22.0,41.0
Texas	3720	15.4(12.3,19.4)	6.0,34.0	34.8(32.7,37)	23.5,49.2	Ohio	2600	14.2(11.9,17.9)	6.5,41.0	36.6(34.3,39.9)		27.5,53.8
Virginia	1595	12.0(9.4,15)	5.5,36.0	34.7(31.9,37.4)	23.5,54.4	South Dakota	179	11(10,13)	6.7,27.8	27.9(26.6,29.4)		22.9,38.2
West Virginia	424	18.8(16.5,21.5)	9.4,35.4	39.8(37.9,42.2)	32.3,50.1	Wisconsin	1263	11.4(10,13.5)	5.8,29.1	30.6(29.1,32.3)		20.1,46.2

*DC: District of Columbia

The US regions were divided based on the US. Census Bureau

#### Exclusion

Florida and Puerto Rico were excluded from the analysis due to inconsistencies in data availability. They did not fully participate in the CDC PLACES program, resulting in missing or incomplete data for key variables (e.g. there is complete missingness of stroke, sleep, and so on for the state of Florida).^24^ Therefore, to maintain the integrity of the analysis, our analysis covered 49 states in the US, and DC.

### Spatial analysis results for the US

**Fig. 1:**
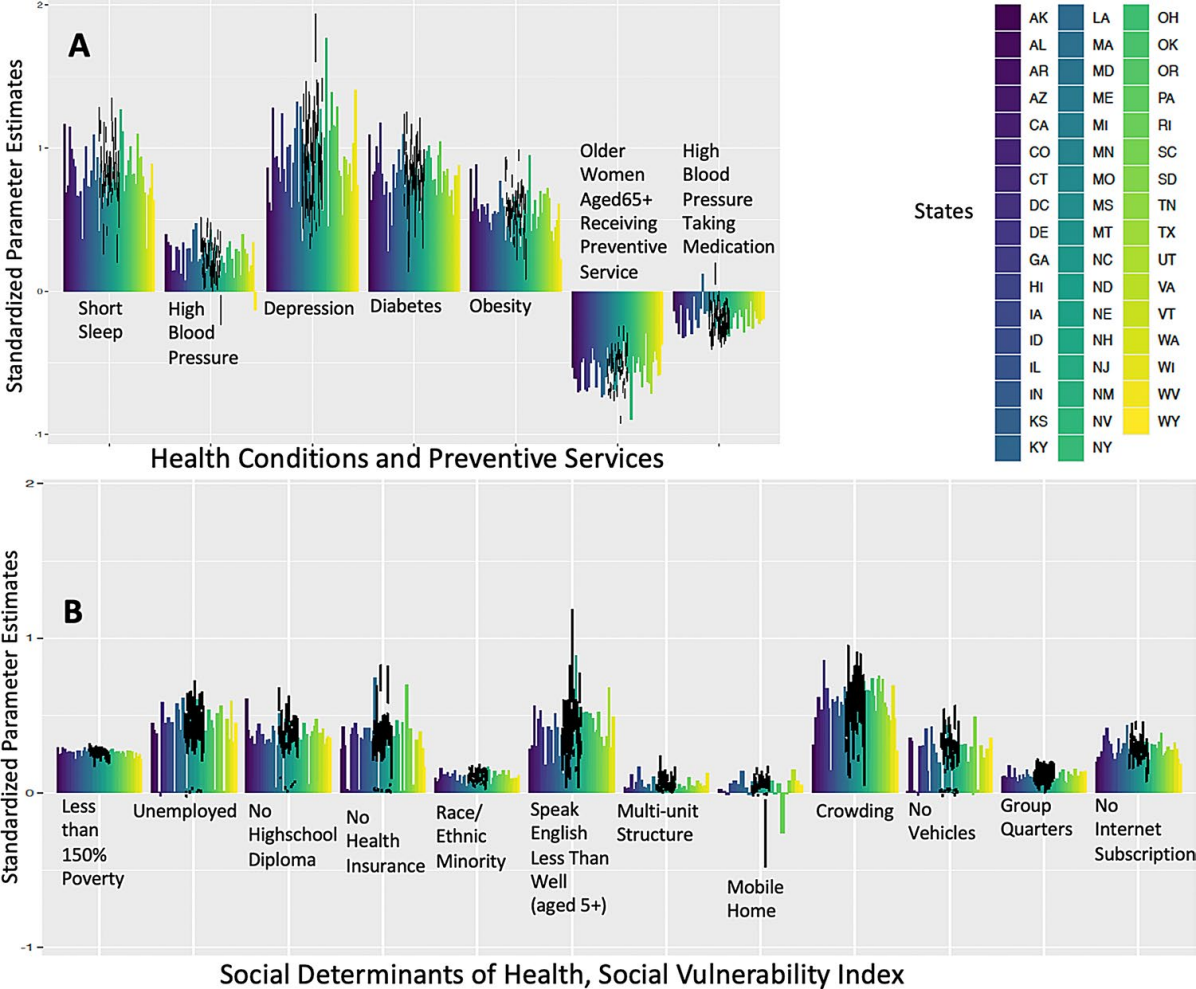
Spatial Model Association with Cognitive Disability adjusting for Short Sleep Duration, Health/Prevention and SoDH/SVI. SoDH: Social Determinants of Health; SVI: Social Vulnerability Index Positive estimates indicate an increase in risk of Cognitive Disability (outcome). Negative estimates indicate an inverse association, meaning they are associated with a decrease in risk of Cognitive Disability (outcome). Definitions of each variable were described in the supplemental material

We conducted a spatial analysis to examine the association between CD and short sleep duration adjusting for other covariates. The results (Fig. 1(A)) revealed a significant association between short sleep duration and an increased risk of CD across the US. The estimate values (i.e. regression coefficient) ranged from 0.29 to 1.27 (*p* < 0.05) in all 50 models, after controlling for other variables. In this spatial analysis, the coefficient represents the direction and strength of the relationship between short sleep and CD. For instance, an estimate of 1.26 in Nevada indicates a 1.26-fold increase in the risk of CD with a one-unit increase in short sleep, or a 126% increase in risk. The strongest relationship between short sleep and CD were observed in three states including New Mexico (NM) with estimate of 1.26 (95%CI = 1.19, 1.34), Alaska with estimate of 1.16 (95%CI = 1.04, 1.28), and Nevada with estimate of 1.11 (95%CI = 1.04, 1.18). On the other hand, the lowest estimate values were found in Vermont with estimate of 0.29 (95%CI = 0.20; 0.39), Hawaii with estimate of 0.36 (95%CI = 0.25; 0.46), and Maryland with estimate of 0.41(95%CI = 0.39; 0.44), indicating that in these states, the relationships between short sleep and CD in these states were much weaker. The detailed estimates for all covariates are provided in Additional Table.

#### Interactive maps of CD and short sleep duration in the US

**Fig. 2:**
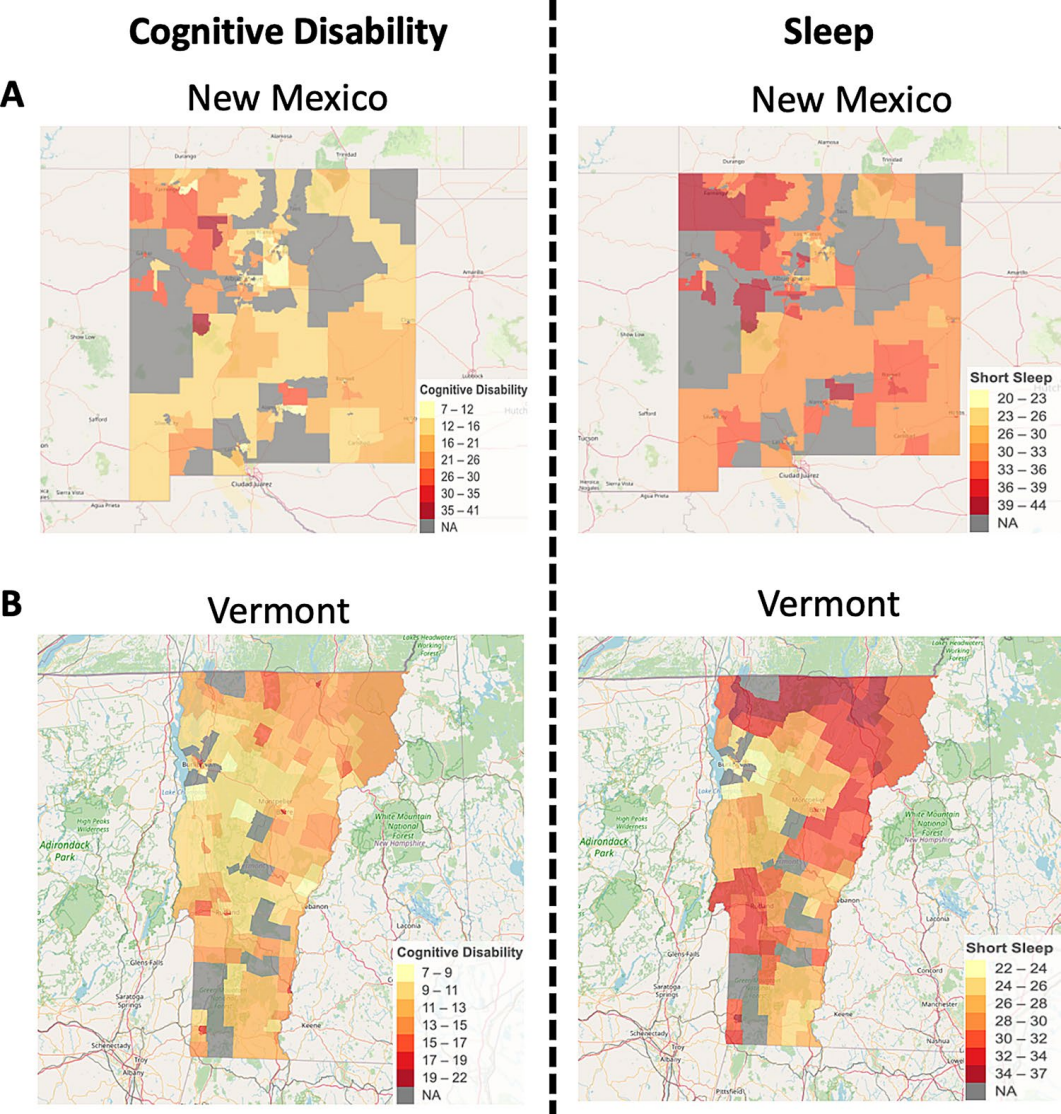
Interactive Maps of Cognitive Disability and Short Sleep Duration in New Mexico and Vermont (The darker, more red areas in both maps represent higher percentages of CD (Figure 2A) or short sleep (Figure 2B))

Among the 49 states and DC, NM and Vermont were selected to illustrate the highest and lowest associations between CD and short sleep duration, as established through spatial analysis. Figure 2 presents interactive maps of CD (Fig. 2A) and short sleep duration (Fig. 2B) for these two states. The darker, more red areas in both maps represent higher percentages of CD (Fig. 2A) or short sleep (Fig. 2B). In Fig. 2A for NM, nearly all of the dark red areas in the CD map indicating high percentage of CD align with areas of high short sleep on the map. This strong overlap reflects the robust association between the two conditions in NM. In contrast, Fig. 2B for Vermont shows a less consistent alignment. Areas with high CD do not always coincide with regions of high short sleep, and several areas with elevated percentage of short sleep do not always align with those having high CD, that suggests a weaker relationship between the two variables in Vermont.

### Explore relationship per region and state

**Fig. 3:**
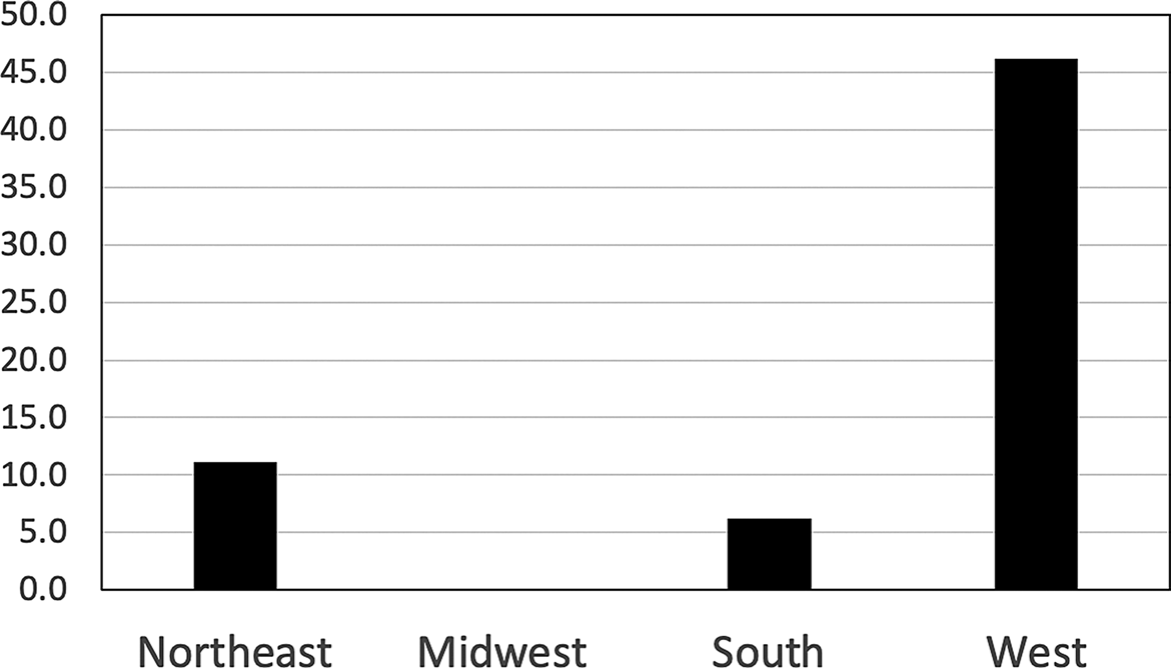
Percentage of States in Each Geographic Region with > 100% increase in risk of Cognitive Disability (*: results obtained from the spatial analysis model adjusting for other confounders)

In Fig. 3, among the 49 states and DC, 8 states exhibited an increase of 100% or more in the risk of CD associated with short sleep duration. Of the 8 states with a risk increase of 100% or more, the majority were in the Western US (Alaska, Oregon, Idaho, Nevada, Arizona, and NM), with South Dakota from the Midwest and Kentucky from the South as the only exceptions. This finding was statistically significant (*p* = 0.007).

#### Analysis weighted by census tract numbers per state

**Fig. 4:**
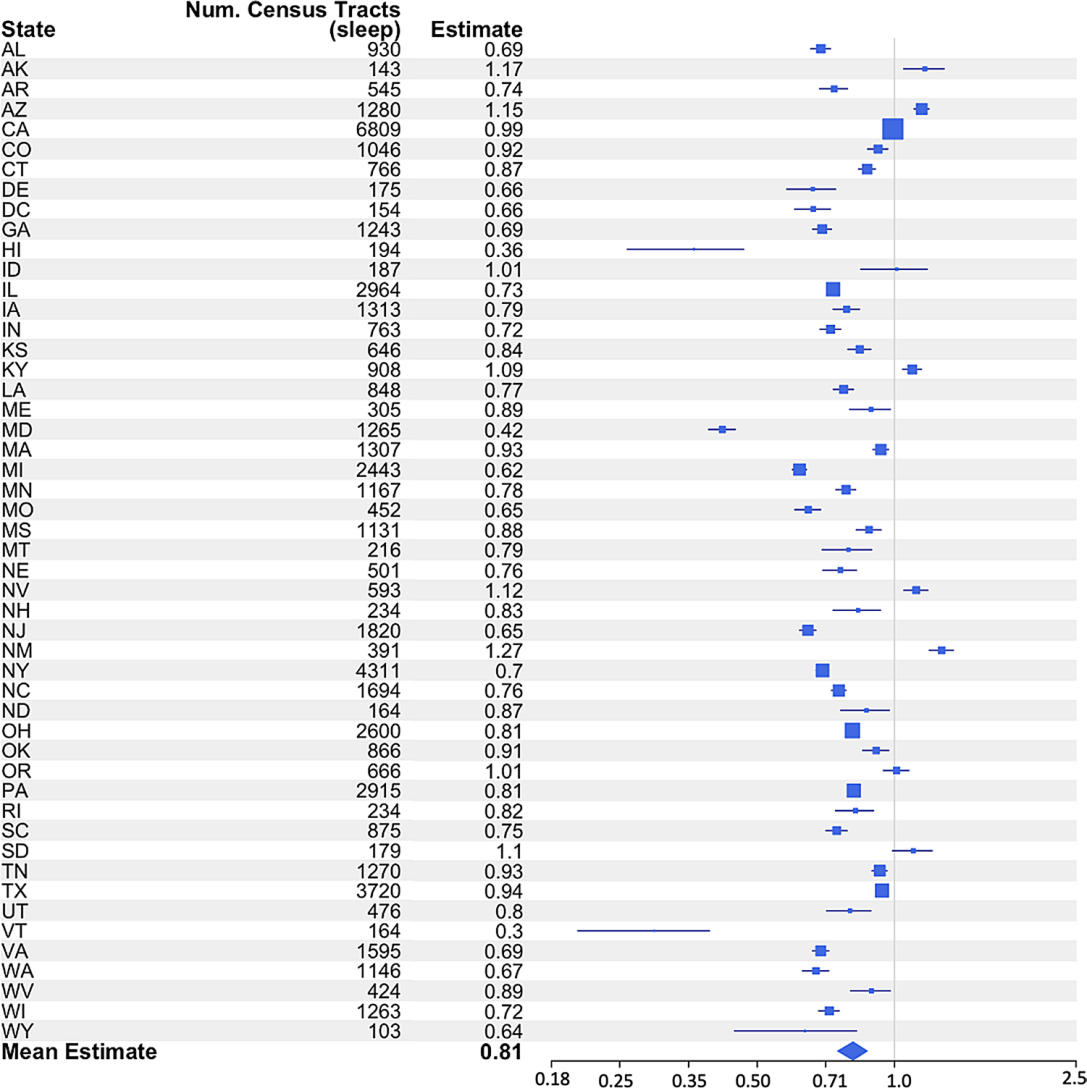
Forest Plot from Analysis Weighted By Census Tract Numbers Per State

The observed effect remained by weighting analysis by the number of census tracts per state to compare regions across the US. The estimate values (i.e. coefficient values) calculated by the analysis weighted by census tract numbers per state, demonstrate a clear geographical gradient in the relationship between short sleep duration and CD. From Fig. 4, it is evident that some states exhibit a considerably higher increased risk of CD due to short sleep duration, with NM and Arizona, for example, showing particularly high estimate (well above 1.0), reflecting an increased risk of more than 100%. This contrasts with states like Vermont and Michigan, where the association between short sleep duration and CD is weaker, with estimates closer to 0.3, suggesting a lower increased risk.

## Discussion

Our cross-sectional spatial analysis at the census-tract level revealed significant associations between short sleep duration and CD across the US. We controlled for common confounder variables reported in many other studies on the relationship between CD and short sleep at the patient-level [34–35]. We found that the estimate value (i.e., regression coefficient) of short sleep duration on CD after adjusting for other confounders ranged from 0.29 (95%CI = 0.20; 0.39) to 1.27 (95%CI = 1.19, 1.34) in 50 US state models in the four regions. Regions with short sleep duration had substantially higher CD risk, consistent with literature that links short sleep duration to an increased CD risk [10- 18, 32–33]. Our unique contribution was adjusting for spatial correlation and other confounder SDoH variables that modulate the relationship between CD and sleep. Those patterns remained strong in the analysis weighted by census-tract numbers and stayed consistently robust across the separate state-level models (excluding Florida and Puerto Rico).

Eight states had CD risk increases of 100% or more associated with short sleep. Six states in the West region (Alaska, Oregon, Idaho, Nevada, Arizona, and New Mexico) had the strongest increase in CD risk associated with short sleep. This highlights the importance of addressing individual health behaviors and broader social and contributing factors such as sleep health, particularly in regions at higher risk for CD.

SDoH includes socioeconomic status, race and ethnicity, education, and geographical location, all of which contribute to disparities in sleep patterns, cognitive health, and other health outcomes [34, 36]. For example, lower-income or marginalized racial groups often face higher levels of chronic stress, fewer resources, and greater barriers to healthcare access, all of which can negatively affect sleep duration [36]. Therefore, when addressing health disparities, geographic differences in health outcomes highlight the importance of understanding how SDoH and geographic patterns affect potential impacts of short sleep on CD. The spatial lag model was selected for this analysis because it captures the direct influence of neighboring regions on the outcome variable [37] —an essential feature when examining the spatial relationship between short sleep duration and CD. By incorporating a spatially lagged dependent variable, the model accounts for potential spillover effects, where outcomes in one region may be influenced by those in adjacent areas. The spatial lag model is better in situations where the outcome is influenced by its neighbors [38]. This characteristic makes the spatial lag model more suitable than alternatives such as the spatial error model (SEM), which addresses spatial autocorrelation in the residuals rather than in the outcome itself [39]. Consequently, the spatial lag model provides a more accurate representation of regional patterns and helps mitigate the risk of biased coefficient estimates and underestimated standard errors. While geographically weighted regression (GWR) allows for spatially varying relationships and can provide localized insights, it is computationally intensive and complex to implement, especially for large datasets [40]. Given these considerations, we selected the spatial lag model as the most appropriate approach for our study.

Our findings on geographic variability align with publications linking short sleep to CD. Ciciora et al [41]. identified short sleep (less than 7 h as a major predictor of Alzheimer’s Disease and Related Dementias (ADRD) at the county level, reinforcing the critical role of sleep and CD. Ciciora’s study used eXtreme Gradient Boosting to rank predictors of ADRD in 3,155 counties. Our assessment of spatial analysis incorporated into geographic variability provides more granular understanding, and our adjustment for several covariates offers more nuance of the short sleep-CD relationship. Ciciora also found that having less than a high school diploma ranked with top ADRD predictors with a mean absolute SHapley Additive exPlanations (SHAP) values of 0.169%. We did not find “less than high school diploma” among the top predictors, but we did find that it increased risk of CD, with a highest estimate of 0.61 (95%CI, 0.57, 0.67) in Alaska and lowest estimate of 0.072 (95%CI, 0.067, 0.077) in Texas. Our finding supports our hypothesis that educational attainment can protect against dementia. Reports including the Framingham Heart Study, [42] Lancet Commission, [43] and a systemic review by Meng and D’Ercy [44] found that education in early life or at least a high school diploma could decrease dementia incidence worldwide. We adjusted for *no high school diploma* in our final geospatial model by considering education as a potential confounder in our spatial analysis, which strengthened our findings’ robustness and precision.

We found that the West had more states having strong associations between short sleep duration and CD, even though it was not the region with the highest levels of inadequate sleep or CD [16]. Causes of the increased risk of CD from short sleep in the West are not immediately clear from our study. While the Midwest and South had higher overall prevalence of CD, this does not necessarily translate to a stronger association between short sleep duration and CD. This apparent discrepancy may reflect regional differences in the distribution of sleep disorders, healthcare access, or reporting practices. Additionally, because our spatial models were adjusted for a range of health and socioeconomic covariates, the observed associations may also be influenced by regional variations in these factors, potentially moderating or attenuating the sleep-CD relationship in certain areas. This regional pattern warrants further investigation. Future research could benefit from exploring different factors such as temperature, noise index, in greater detail.

Our study has several strengths. It provides a unique contribution by conducting a detailed geospatial analysis at the census tract level, offering a granular view of the geographic variability in the relationship between short sleep duration and CD across the entire US. Additionally, by adjusting for multiple covariates, including several known potential confounders, we were able to provide a nuanced interpretation of the short sleep-CD relationship. The large sample size, covering the entire US, strengthens the generalizability of our findings and provides more robust estimates of risk. Furthermore, the state-level weighted analysis, which accounts for the number of census tracts in each state, improves the precision of our results, and offers a novel way to visualize how short sleep duration affects CD across different regions of the US.

Still our study has several limitations. The cross-sectional design restricts causality establishment and recall bias of short sleep self-reporting may also affect data accuracy. We adjusted for 18 covariates, but there may still be unmeasured confounders—such as genetic factors or individual sleep quality—that could influence the relationship. Lack of longitudinal data prevents assessment of how sleep duration changes over time might impact CD, and while our census-tract level data provides fine-grained analysis, it does not account for other potential neighborhood-level factors such as environmental influences. Further research could benefit from a longitudinal approach to better assess causality and explore how changes in sleep duration over time may contribute to CD onset and progression. Using more objective measures of sleep duration such as actigraphy or polysomnography could provide more accurate data to address recall bias.

## Conclusion

We align with the literature on sleep in CD, but are among the first to examine it across the US. Our strongest associations were in the West, a novel regional insight. We emphasize the critical need to consider sleep health as an important protector of individual behaviors and broader social determinants of cognitive health disparities, particularly in regions at higher risk for CD. Future studies should adopt longitudinal designs to clarify causal pathways and evaluate how temporal changes in sleep duration influence the development and progression of CD.

## Electronic supplementary material

Below is the link to the electronic supplementary material.

Supplementary Material 1

## Data Availability

No datasets were generated or analysed during the current study.
