# Supplementary material for: Geospatial analysis of short sleep duration and cognitive disability in US adults: a multi-state study using machine learning techniques
Source: BioData Min. 2025 Jun 13;18:41. doi: 10.1186/s13040-025-00456-7 (PMC12166631; doi:10.1186/s13040-025-00456-7)
Supplement: Supplementary file 1 — Supplementary Material 1 [file 13040_2025_456_MOESM1_ESM.docx]

**Geospatial Analysis of Short Sleep Duration and Cognitive Disability in US Adults:**

**A Multi-State Study Using Machine Learning Techniques**

Tue T. Te, MD, MBMI^1,2^, Alex A.T. Bui, PhD^1,3^, Constance H. Fung, MD, MSHS^1,2^, Mary Regina Boland, MA, MPhil, PhD, FAMIA^4^

1. Department of Medicine, David Geffen School of Medicine at University of California, Los Angeles (UCLA), CA, USA.
2. Geriatric, Research, Education and Clinical Center, VA Greater Los Angeles, Los Angeles, CA, USA.
3. Medical and Imaging Informatics Group, Department of Radiological Sciences, University of California, Los Angeles, Los Angeles, California, USA.
4. Department of Data Science, Mathematics, Herbert W. Boyer School of Natural Sciences, Mathematics, and Computing, Alex G McKenna School of Business, Economics and Government, Saint Vincent College, Latrobe, PA, USA.

Corresponding author: Tue Te, MD. Address: 1260 15^th^ Street, Suite 600, Santa Monica, CA 90404, California, USA. Email: TTe@mednet.ucla.edu. Tel: 310-449-0939. Fax: 424-259-7790.

Institution where the work was performed: University of California, Los Angeles, CA, USA

Additional Methods 2

Additional Results 3

Additional Discussion 3

Additional Table. Spatial Analysis Results for the US 4

Additional Table. Spatial Analysis Results for the US (continue) 5

Additional Table. Spatial Analysis Results for the US (continue) 6

Additional Table. Spatial Analysis Results for the US (continue) 7

Additional Table. Spatial Analysis Results for the US (continue) 8

**ADDITIONAL METHODS**

**Dataset**

**CDC PLACES** provides detailed information on health outcomes, prevention practices, health risk behaviors, disabilities, health statuses, and community factors, which are collected from national surveys such as the Behavioral Risk Factor Surveillance System (BRFSS). For instance, CD was defined as: "Because of a physical, mental, or emotional condition, do you have serious difficulty concentrating, remembering, or making decisions?".^21^ Short sleep duration, or sleep of less than 7 hours, was assessed by asking individuals: "How many hours of sleep do you get on average in a 24-hour period?”.^21^ These data are available at the census tract levels across the US and their percentages of each health condition were already computed using a multi-level regression, post-stratification approach based on BRFSS and American Community Survey (ACS) data. We extracted the estimated percentages of each health condition available in the CDC PLACES dataset, focusing on those with minimal missing data across all counties and census tracts nationwide. Package *CDCPLACES* (version 1.1.8) in R (version 4.3.3) was used.

**Variables**: The dataset used for analysis includes a variety of health, socioeconomic, and demographic variables that are critical to understanding the relationships between CD and other predictors, such as sleep duration. The outcome variable of primary interest is the cognitive disability, and the main predictor variable is the short sleep duration. In addition to these, our dataset also includes 18 other variables across several domains: 1) Health outcomes includes percentage of persons with high blood pressure estimate, percentage of persons with diabetes estimate, percentage of persons with depression estimate, percentage of persons with obesity estimate; 2) Preventive services includes percentage of persons with high blood pressure taking medications estimate, percentage of older woman aged 65+ receiving prevention service estimate; 3) Race/ethnic minority status includes percentage minority (Hispanic or Latino (of any race); Black and African American, Not Hispanic or Latino; American Indian and Alaska Native, Not Hispanic or Latino; Asian, Not Hispanic or Latino; Native Hawaiian and Other Pacific Islander, Not Hispanic or Latino; Two or More Races, Not Hispanic or Latino; Other Races, Not Hispanic or Latino) estimate; 4) Socioeconomic status includes percentage of persons below 150% poverty estimate, percentage of persons with unemployment estimate, percentage of persons with no high school diploma with aged 25+ estimate, percentage of persons with no health insurance estimate, percentage of persons speaking English “less than well” with aged 5+ estimate; 5) Housing and transportation includes percentage of housing in structures with 10 or more units estimate (multi-unit structure), percentage of persons in mobile homes estimate (mobile home), percentage of households with more people than rooms estimate (crowded), percentage of persons in group quarters estimate (group quarter), percentage of households with no vehicles estimate, percentage of households without an internet subscription estimate.

**CDC SVI** is a tool developed to assess the vulnerability of communities to various hazards. It uses US Census data to evaluate factors such as income, education, employment, housing quality, transportation access, and racial/ethnic composition, assigning a score to each census tract to indicate its level of social vulnerability.^22^ Similarly, we retrieved the health condition estimates from the CDC SVI, selecting only those with the least amount of missing data for comprehensive coverage at the county and census-tract levels. Package CDCPLACES (version 1.1.8) in R (version 4.3.3) was used.

**Shapefiles:** The 2010 geographic shapefiles for 50 US states including District of Columbia’ census tracts were downloaded from the US Census Bureau's Cartographic Boundary Files and used to map and visualize the census tract-level data. ^23^ All shapefiles were read into R (version 4.3.3) using the *sf* package (version 1.0-18), and census tracts were imported into a spatial data frame for further analysis.

**Spatial Analysis**

**Spatial weight matrix construction.** S*pdep* (version 1.3-6)^26^ and *spatialreg* (version 1.3-5) ^27^ packages in R were used. A spatial adjacency matrix was created using neighborhood information derived from shapefiles, which constructs a spatial weight matrix based on contiguity. The adjacency of tracts was determined by identifying neighboring polygons based on shared boundaries. The matrix was then symmetrized by adding its transpose and subtracting any self-links. We set *true* for *zero policy* option to account for census tracts that have no neighbors, ensuring that all tracts were included in the model.

**Spatial analysis model specification.** The *spatial lag* models were constructed separately for each state, therefore we conducted 50 models for 50 states. These models incorporated the lagged values of neighboring tracts' CD as the outcome, short sleep duration as a main predictor, adjusting for covariates from the CDC PLACES in prevalence of health outcomes, prevention practices, health risk behaviors, disabilities, health statuses, and community factors, also from the CDC SVI in socio-economic and income, education, employment, housing quality, transportation access, and racial/ethnic composition factors within each state. The coefficients, p-values, and confidence intervals (CI) were extracted from these models (p-value<0.05 is considered significant).

**State-Level Weighted Forest Plot**. We weighted our analysis by the number of census-tracts per state as well to generate a state-level weighted result. The mean estimates for each state were incorporated into forest plot visualizations. The weighted estimates were also summarized at the national level with computed mean estimate across all states.

**Choropleth-like visualization**. We visualized the relationships between CD and short sleep duration along with various health and socio-economic variables across US states using choropleth and heatmap techniques. We plotted the estimates and CIs for the state with the highest estimate (risk of cognitive disability) and the state with the lowest estimate (lowest risk of cognitive disability) displaying all health conditions and socio-economic factors using *ggplot2* package (version 3.5.1)^28^ in R.

**ADDITIONAL RESULTS**

Overview of dataset and variables

**Dataset Overview.** The dataset divides the US into four regions: Northeast (9 states), Midwest (12 states), South (17 states), and West (13 states). In Table 1, California has the most census tracts (6,809), followed by New York (4,311) and Texas (3,720). The states with the fewest census tracts are Wyoming (103), Alaska (143), and the District of Columbia (157). Regarding cognitive disability, the South and Midwest regions tend to have higher percentages with Mississippi reports the highest median at 19.4% (IQR: 16.6–22.9%), followed by Louisiana at 18.9% (IQR: 15.4–22.2%); in contrast, Hawaii has the lowest median at 9.7% (IQR: 8.1–11.1%), followed by Nebraska at 10.3% (IQR: 9.0–12.1%). For the short sleep duration, Hawaii has the highest median at 39.85% (IQR: 37.6–42.2%), followed closely by West Virginia at 39.75% (IQR: 37.9–42.2%). States with the lowest medians include South Dakota at 27.9% (IQR: 26.6–29.4%) and Minnesota at 28.0% (IQR: 25.8–29.8%).

**Variables.** The dataset used for analysis includes a variety of health, socioeconomic, and demographic variables that are critical to understanding the relationships between CD and other predictors, such as sleep duration. The outcome variable of primary interest is the *cognitive disability*, and the main predictor variable is the *short sleep duration*. In addition to these, our dataset also includes 18 other variables across several domains including Health outcomes, Preventive services, Race/ethnic minority status, SES, Housing and transportation.

**Spatial Analysis Results for the US**

In addition, preventive services were found to reduce the risk of cognitive disability, while health conditions such as diabetes and depression were consistently associated with an increased risk across all states. High blood pressure was generally linked to an increased risk of cognitive disability, but it was not statistically significant in several states, including Arizona, Hawaii, Idaho, North Dakota, Rhode Island, Utah, and Vermont. On the other hand, preventive services, such as the use of medications for high blood pressure and preventive care for older women, mitigated the risk of cognitive disability, with older women receiving preventive services showing a consistently reduced risk across all states. However, the use of high blood pressure medications did not significantly reduce CD risk in several states, including Alabama, Arkansas, the District of Columbia, Georgia, Maine, Mississippi, Tennessee, and Virginia. Social determinants of health (SDoH) were generally found to increase the risk of CD - Figure 1(B), with poverty below 150% of the federal poverty line and lack of internet subscription being significant in the US. Poverty estimates ranged from 0.19 in Hawaii to 0.29 in Arizona, while the lack of internet subscription ranged from 0.12 in Vermont to 0.43 in Massachusetts. Other SDoH covariates were significant in the majority of states, but their effects were not consistent across the US.

**eDISCUSSION**

Additionally, Ma et al.^34^ explored disparities in access to sleep health services and their impact on cognitive difficulties, particularly in underserved communities across the US. Using rural-urban commuting area codes and the 2020 Area Deprivation Index, Ma and colleagues found that limited access to sleep health services exacerbated cognitive difficulties, especially in disadvantaged areas. Our study did not directly address rural-urban or disadvantage area differences; but we modelled spatial variability at the census-tract (neighborhood) level within each state which accounts for regional variation in the states. We identified a notable pattern in the Western US, where short sleep duration was associated with a more than 100% increase in the risk of cognitive disability, even after controlling for SDoH and SVI covariates.

**ADDITIONAL TABLE. Spatial Analysis Results for the US**

| Variables | Alabama | Alaska | Arkansas | Arizona | California | Colorado | Connecticut | Delaware | District of Columbia | Georgia |
| --- | --- | --- | --- | --- | --- | --- | --- | --- | --- | --- |
| Short Sleep | 0.69 | 1.17 | 0.74 | 1.15 | 0.99 | 0.92 | 0.87 | 0.66 | 0.66 | 0.69 |
| Older Women Aged 65+ Receiving Preventive Services | -0.61 | -0.53 | -0.61 | -0.71 | -0.69 | -0.49 | -0.47 | -0.70 | -0.68 | -0.67 |
| Depression | 0.62 | 0.86 | 0.56 | 1.28 | 0.93 | 0.94 | 0.86 | 1.24 | 0.76 | 0.85 |
| Obesity | 0.56 | 0.85 | 0.65 | 0.88 | 0.57 | 0.48 | 0.60 | 0.53 | 0.59 | 0.61 |
| Diabetes | 0.64 | 1.09 | 0.81 | 0.83 | 1.01 | 0.90 | 1.18 | 0.67 | 0.82 | 0.76 |
| High Blood Pressure | 0.34 | 0.39 | 0.32 | NA* | 0.14 | 0.15 | 0.31 | 0.12 | 0.28 | 0.34 |
| Less than 150% Poverty | 0.25 | 0.29 | 0.25 | 0.29 | 0.28 | 0.26 | 0.27 | 0.26 | 0.27 | 0.26 |
| Unemployed | 0.40 | 0.45 | 0.38 | NA* | 0.01 | -0.02 | 0.59 | 0.37 | 0.45 | NA* |
| No Highschool Diploma Aged 25+ | 0.40 | 0.61 | 0.35 | 0.36 | 0.10 | 0.31 | 0.40 | 0.32 | 0.44 | 0.38 |
| No Health Insurance | 0.42 | 0.28 | 0.31 | 0.03 | 0.02 | 0.38 | 0.42 | 0.38 | 0.45 | 0.02 |
| Speak English Less Than Well Aged 5+ | 0.28 | NA* | 0.31 | 0.56 | 0.30 | 0.47 | 0.53 | 0.42 | NA* | 0.18 |
| Minority | 0.08 | 0.10 | 0.09 | 0.16 | 0.09 | 0.12 | 0.12 | 0.11 | 0.13 | 0.09 |
| Mobile Homes | 0.03 | NA* | NA* | 0.01 | NA* | -0.01 | NA* | NA* | NA* | NA* |
| Crowding | 0.48 | 0.31 | 0.49 | 0.62 | 0.31 | 0.54 | 0.86 | 0.67 | 0.30 | 0.54 |
| No Vehicles | 0.36 | 0.01 | 0.33 | 0.02 | 0.01 | -0.01 | 0.30 | 0.24 | NA* | NA* |
| Group Quarters | 0.11 | NA* | 0.11 | 0.10 | 0.07 | 0.18 | 0.09 | 0.16 | 0.10 | 0.10 |
| No Internet Subscription | 0.23 | 0.20 | 0.19 | 0.27 | 0.37 | 0.32 | 0.42 | 0.31 | 0.33 | 0.26 |
| High Blood Pressure Taking Medication | NA* | -0.13 | NA* | -0.22 | -0.30 | -0.10 | -0.32 | -0.31 | NA* | NA* |
| Multi-unit Structure | NA* | NA* | NA* | 0.04 | 0.03 | 0.03 | 0.12 | NA* | NA* | 0.03 |

**: this variable is not statistically significant in the given model.*

**ADDITIONAL TABLE. Spatial Analysis Results for the US (continue)**

| Variables | Hawaii | Idaho | Illinois | Iowa | Indiana | Kansas | Kentucky | Louisiana | Maine | Maryland |
| --- | --- | --- | --- | --- | --- | --- | --- | --- | --- | --- |
| Short Sleep | 0.36 | 1.01 | 0.73 | 0.79 | 0.72 | 0.84 | 1.09 | 0.77 | 0.89 | 0.42 |
| Older Women Aged 65+ Receiving Preventive Services | -0.50 | -0.48 | -0.67 | -0.40 | -0.49 | -0.53 | -0.74 | -0.72 | -0.52 | -0.39 |
| Depression | 0.69 | 1.02 | 0.59 | 1.00 | 0.90 | 1.14 | 1.32 | 0.62 | 1.03 | 0.35 |
| Obesity | 0.48 | 0.54 | 0.56 | 0.42 | 0.54 | 0.55 | 0.77 | 0.65 | 0.45 | 0.35 |
| Diabetes | 0.28 | 0.46 | 0.88 | 0.69 | 0.72 | 0.83 | 0.99 | 0.85 | 0.83 | 0.72 |
| High Blood Pressure | NA* | NA* | 0.30 | 0.08 | 0.30 | 0.21 | 0.43 | 0.47 | 0.26 | 0.22 |
| Less than 150% Poverty | 0.19 | 0.27 | 0.27 | 0.22 | 0.24 | 0.27 | 0.29 | 0.27 | 0.24 | 0.25 |
| Unemployed | NA* | 0.45 | 0.45 | 0.49 | 0.01 | 0.58 | 0.54 | 0.41 | 0.37 | NA* |
| No Highschool Diploma Aged 25+ | NA* | 0.29 | 0.34 | 0.31 | 0.34 | 0.33 | 0.43 | 0.40 | 0.51 | 0.30 |
| No Health Insurance | NA* | 0.35 | 0.42 | 0.35 | 0.30 | 0.42 | 0.33 | 0.40 | 0.29 | NA* |
| Speak English Less Than Well Aged 5+ | 0.10 | 0.40 | 0.19 | 0.43 | 0.52 | 0.42 | 0.24 | NA* | 0.52 | 0.13 |
| Minority | NA* | 0.15 | 0.08 | 0.13 | 0.08 | 0.12 | 0.06 | 0.11 | 0.12 | 0.04 |
| Mobile Homes | NA* | 0.06 | 0.08 | NA* | NA* | 0.06 | 0.14 | 0.03 | 0.07 | NA* |
| Crowding | 0.15 | 0.58 | 0.59 | 0.60 | 0.62 | 0.50 | 0.58 | 0.57 | 0.43 | 0.35 |
| No Vehicles | NA* | 0.41 | 0.14 | 0.30 | 0.01 | 0.43 | 0.32 | 0.26 | 0.20 | 0.03 |
| Group Quarters | 0.07 | 0.09 | 0.12 | 0.11 | 0.10 | 0.15 | 0.12 | 0.11 | 0.12 | 0.10 |
| No Internet Subscription | 0.21 | 0.26 | 0.35 | 0.22 | 0.26 | 0.28 | 0.32 | 0.25 | 0.26 | 0.28 |
| High Blood Pressure Taking Medication | -0.12 | -0.30 | -0.04 | -0.20 | -0.20 | -0.25 | -0.12 | 0.12 | NA* | -0.14 |
| Multi-unit Structure | NA* | 0.17 | NA* | 0.07 | 0.08 | 0.04 | NA* | 0.03 | 0.11 | 0.01 |

**: this variable is not statistically significant in the given model.*

**ADDITIONAL TABLE. Spatial Analysis Results for the US (continue)**

| Variables | Massachusetts | Michigan | Minnesota | Missouri | Mississippi | Montana | Nebraska | Nevada | New Hampshire | New Jersey |
| --- | --- | --- | --- | --- | --- | --- | --- | --- | --- | --- |
| Short Sleep | 0.93 | 0.62 | 0.78 | 0.65 | 0.88 | 0.79 | 0.76 | 1.12 | 0.83 | 0.65 |
| Older Women Aged 65+ Receiving Preventive Services | -0.60 | -0.56 | -0.46 | -0.69 | -0.70 | -0.48 | -0.28 | -0.90 | -0.46 | -0.55 |
| Depression | 1.29 | 0.62 | 0.98 | 0.95 | 0.59 | 0.74 | 0.80 | 1.77 | 1.27 | 0.56 |
| Obesity | 0.59 | 0.52 | 0.34 | 0.59 | 0.66 | 0.54 | 0.44 | 0.95 | 0.61 | 0.54 |
| Diabetes | 1.10 | 0.76 | 0.78 | 0.85 | 0.80 | 0.86 | 0.65 | 1.01 | 0.86 | 0.92 |
| High Blood Pressure | 0.21 | 0.30 | 0.14 | 0.36 | 0.43 | 0.12 | 0.11 | 0.13 | 0.11 | 0.25 |
| Less than 150% Poverty | 0.28 | 0.24 | 0.24 | 0.29 | 0.26 | 0.25 | 0.22 | 0.28 | 0.27 | 0.25 |
| Unemployed | 0.61 | 0.03 | NA* | 0.56 | 0.39 | 0.47 | NA* | 0.40 | 0.40 | 0.39 |
| No Highschool Diploma Aged 25+ | 0.03 | 0.43 | 0.01 | 0.48 | 0.43 | 0.54 | 0.30 | 0.34 | 0.38 | 0.35 |
| No Health Insurance | 0.74 | 0.02 | NA* | 0.46 | 0.43 | 0.27 | NA* | 0.47 | 0.43 | 0.33 |
| Speak English Less Than Well Aged 5+ | 0.37 | 0.31 | 0.47 | 0.42 | NA* | NA* | 0.45 | 0.52 | 0.55 | 0.26 |
| Minority | 0.12 | 0.08 | 0.09 | 0.07 | 0.11 | 0.13 | 0.12 | 0.15 | 0.13 | 0.08 |
| Mobile Homes | -0.01 | NA* | NA* | 0.08 | NA* | NA* | NA* | 0.08 | NA* | NA* |
| Crowding | 0.68 | 0.69 | 0.54 | 0.65 | 0.65 | 0.51 | 0.70 | 0.66 | 0.72 | 0.41 |
| No Vehicles | NA* | 0.02 | 0.01 | 0.33 | 0.44 | 0.37 | NA* | 0.31 | 0.33 | 0.18 |
| Group Quarters | 0.07 | 0.11 | 0.16 | 0.14 | 0.11 | 0.13 | 0.05 | 0.09 | 0.15 | 0.10 |
| No Internet Subscription | 0.43 | 0.30 | 0.28 | 0.31 | 0.20 | 0.25 | 0.25 | 0.32 | 0.33 | 0.32 |
| High Blood Pressure Taking Medication | -0.16 | -0.18 | -0.15 | -0.08 | NA* | -0.26 | -0.11 | -0.26 | -0.28 | -0.16 |
| Multi-unit Structure | 0.06 | 0.05 | 0.04 | NA* | 0.07 | 0.10 | 0.04 | 0.10 | 0.05 | 0.04 |

**: this variable is not statistically significant in the given model.*

**ADDITIONAL TABLE. Spatial Analysis Results for the US (continue)**

| Variables | New  Mexico | New York | North Carolina | North Dakota | Ohio | Oklahoma | Oregon | Pennsylvania | Rhode Islands | South Carolina |
| --- | --- | --- | --- | --- | --- | --- | --- | --- | --- | --- |
| Short Sleep | 1.27 | 0.70 | 0.76 | 0.87 | 0.81 | 0.91 | 1.01 | 0.81 | 0.82 | 0.75 |
| Older Women Aged 65+ Receiving Preventive Services | -0.57 | -0.45 | -0.54 | -0.42 | -0.57 | -0.50 | -0.46 | -0.56 | -0.67 | -0.52 |
| Depression | 1.07 | 0.45 | 0.88 | 1.09 | 1.12 | 1.39 | 1.16 | 1.15 | 1.29 | 0.84 |
| Obesity | 0.70 | 0.42 | 0.61 | 0.31 | 0.54 | 0.64 | 0.44 | 0.52 | 0.70 | 0.55 |
| Diabetes | 0.98 | 0.93 | 0.77 | 0.54 | 0.77 | 0.78 | 0.67 | 0.95 | 1.05 | 0.69 |
| High Blood Pressure | 0.20 | 0.31 | 0.31 | NA* | 0.35 | 0.23 | 0.09 | 0.29 | NA* | 0.27 |
| Less than 150% Poverty | 0.28 | 0.25 | 0.27 | 0.23 | 0.26 | 0.27 | 0.27 | 0.26 | 0.27 | 0.26 |
| Unemployed | 0.37 | 0.01 | 0.47 | 0.60 | 0.54 | 0.47 | 0.42 | 0.02 | 0.51 | 0.40 |
| No Highschool Diploma Aged 25+ | 0.36 | 0.04 | 0.41 | 0.34 | 0.45 | 0.37 | 0.38 | 0.06 | 0.36 | 0.40 |
| No Health Insurance | 0.36 | NA* | 0.39 | 0.29 | 0.38 | 0.01 | 0.45 | 0.02 | 0.70 | 0.34 |
| Speak English Less Than Well Aged 5+ | 0.52 | 0.15 | 0.38 | 0.89 | 0.51 | 0.48 | 0.39 | 0.40 | 0.52 | 0.36 |
| Minority | 0.17 | 0.06 | 0.09 | 0.13 | 0.10 | 0.14 | 0.11 | 0.09 | 0.12 | 0.11 |
| Mobile Homes | 0.08 | -0.01 | 0.07 | 0.09 | NA* | NA* | 0.06 | NA* | -0.26 | -0.01 |
| Crowding | 0.66 | 0.23 | 0.65 | 0.69 | 0.74 | 0.63 | 0.53 | 0.74 | 0.76 | 0.65 |
| No Vehicles | 0.21 | 0.00 | 0.02 | 0.30 | 0.31 | NA* | 0.20 | 0.02 | 0.30 | -0.01 |
| Group Quarters | 0.08 | 0.09 | 0.12 | 0.07 | 0.13 | 0.11 | 0.15 | 0.08 | 0.07 | 0.14 |
| No Internet Subscription | 0.21 | 0.27 | 0.28 | 0.24 | 0.31 | 0.24 | 0.29 | 0.33 | 0.39 | 0.24 |
| High Blood Pressure Taking Medication | -0.32 | -0.18 | -0.03 | -0.23 | -0.15 | -0.26 | -0.13 | -0.18 | -0.29 | -0.12 |
| Multi-unit Structure | NA* | 0.02 | NA* | 0.04 | 0.06 | 0.03 | 0.04 | 0.02 | 0.10 | NA* |

**: this variable is not statistically significant in the given model.*

| Variables | South Dakota | Tennessee | Texas | Utah | Vermont | Virginia | Washington | West Virginia | Wisconsin | Wyoming |
| --- | --- | --- | --- | --- | --- | --- | --- | --- | --- | --- |
| Short Sleep | 1.10 | 0.93 | 0.94 | 0.80 | 0.30 | 0.69 | 0.67 | 0.89 | 0.72 | 0.64 |
| Older Women Aged 65+ Receiving Preventive Services | -0.29 | -0.63 | -0.64 | -0.71 | -0.42 | -0.49 | -0.48 | -0.58 | -0.59 | -0.37 |
| Depression | 0.80 | 0.53 | 0.94 | 0.92 | 0.89 | 0.81 | 0.70 | 1.41 | 1.03 | 0.74 |
| Obesity | 0.68 | 0.68 | 0.72 | 0.65 | 0.35 | 0.42 | 0.45 | 0.61 | 0.50 | 0.22 |
| Diabetes | 0.84 | 0.81 | 0.85 | 0.66 | 0.53 | 0.71 | 0.81 | 0.88 | 0.81 | NA* |
| High Blood Pressure | 0.11 | 0.40 | 0.29 | NA* | NA* | 0.26 | 0.14 | 0.34 | 0.18 | -0.13 |
| Less than 150% Poverty | 0.23 | 0.27 | 0.27 | 0.26 | 0.22 | 0.27 | 0.26 | 0.25 | 0.24 | 0.22 |
| Unemployed | 0.56 | 0.01 | 0.01 | NA* | 0.35 | 0.48 | NA* | 0.33 | 0.60 | 0.45 |
| No Highschool Diploma Aged 25+ | 0.43 | 0.48 | 0.07 | 0.39 | 0.41 | 0.39 | 0.31 | 0.37 | 0.35 | 0.35 |
| No Health Insurance | 0.41 | 0.01 | 0.05 | NA* | NA* | 0.34 | 0.01 | 0.29 | 0.40 | 0.17 |
| Speak English Less Than Well Aged 5+ | 0.26 | 0.30 | 0.36 | 0.68 | NA* | NA* | 0.29 | NA* | 0.49 | NA* |
| Minority | 0.15 | 0.07 | 0.11 | 0.15 | 0.09 | 0.05 | 0.07 | 0.07 | 0.10 | 0.11 |
| Mobile Homes | 0.08 | NA* | NA* | NA* | NA* | 0.15 | NA* | 0.08 | NA* | 0.05 |
| Crowding | 0.74 | 0.58 | 0.55 | 0.50 | NA* | 0.36 | 0.47 | 0.48 | 0.69 | 0.27 |
| No Vehicles | 0.49 | 0.02 | 0.01 | NA* | 0.23 | 0.30 | 0.01 | 0.24 | 0.28 | 0.36 |
| Group Quarters | 0.12 | 0.11 | 0.08 | 0.16 | 0.11 | 0.11 | 0.14 | 0.14 | 0.12 | 0.14 |
| No Internet Subscription | 0.25 | 0.27 | 0.30 | 0.27 | 0.12 | 0.28 | 0.32 | 0.22 | 0.28 | 0.19 |
| High Blood Pressure Taking Medication | -0.25 | NA* | -0.07 | -0.26 | -0.14 | NA* | -0.19 | -0.20 | -0.23 | -0.19 |
| Multi-unit Structure | 0.05 | 0.04 | 0.01 | 0.08 | 0.06 | NA* | 0.04 | 0.13 | 0.05 | NA* |

**ADDITIONAL TABLE. Spatial Analysis Results for the US (continue)**

**: this variable is not statistically significant in the given model.*
